# Development and application of triple antibody sandwich enzyme-linked immunosorbent assays for begomovirus detection using monoclonal antibodies against *Tomato yellow leaf curl Thailand virus*

**DOI:** 10.1186/s12985-017-0763-z

**Published:** 2017-05-30

**Authors:** Channarong Seepiban, Saengsoon Charoenvilaisiri, Nuchnard Warin, Anjana Bhunchoth, Namthip Phironrit, Bencharong Phuangrat, Orawan Chatchawankanphanich, Supat Attathom, Oraprapai Gajanandana

**Affiliations:** 1 Virology and Antibody Technology Research Unit, National Center for Genetic Engineering and Biotechnology (BIOTEC), National Science and Technology Development Agency (NSTDA), 113 Thailand Science Park, Phahonyothin Road, Klong Nueng, Klong Luang, Pathum Thani 12120 Thailand; 2 Department of Plant Pathology, Faculty of Agriculture Kamphaeng Saen, Kasetsart University, Kamphaeng Saen Campus, Nakhon Pathom, 73140 Thailand

**Keywords:** *Tomato yellow leaf curl Thailand virus*, Begomovirus, Coat protein, Monoclonal antibody, TAS-ELISA

## Abstract

**Background:**

*Tomato yellow leaf curl Thailand virus*, TYLCTHV, is a begomovirus that causes severe losses of tomato crops in Thailand as well as several countries in Southeast and East Asia. The development of monoclonal antibodies (MAbs) and serological methods for detecting TYLCTHV is essential for epidemiological studies and screening for virus-resistant cultivars.

**Methods:**

The recombinant coat protein (CP) of TYLCTHV was expressed in *Escherichia coli* and used to generate MAbs against TYLCTHV through hybridoma technology. The MAbs were characterized and optimized to develop triple antibody sandwich enzyme-linked immunosorbent assays (TAS-ELISAs) for begomovirus detection. The efficiency of TAS-ELISAs for begomovirus detection was evaluated with tomato, pepper, eggplant, okra and cucurbit plants collected from several provinces in Thailand. Molecular identification of begomoviruses in these samples was also performed through PCR and DNA sequence analysis of the CP gene.

**Results:**

Two MAbs (M1 and D2) were generated and used to develop TAS-ELISAs for begomovirus detection. The results of begomovirus detection in 147 field samples indicated that MAb M1 reacted with 2 begomovirus species, TYLCTHV and *Tobacco leaf curl Yunnan virus* (TbLCYnV), whereas MAb D2 reacted with 4 begomovirus species, TYLCTHV, TbLCYnV, *Tomato leaf curl New Delhi virus* (ToLCNDV) and *Squash leaf curl China virus* (SLCCNV). Phylogenetic analyses of CP amino acid sequences from these begomoviruses revealed that the CP sequences of begomoviruses recognized by the narrow-spectrum MAb M1 were highly conserved, sharing 93% identity with each other but only 72–81% identity with MAb M1-negative begomoviruses. The CP sequences of begomoviruses recognized by the broad-spectrum MAb D2 demonstrated a wider range of amino acid sequence identity, sharing 78–96% identity with each other and 72–91% identity with those that were not detected by MAb D2.

**Conclusions:**

TAS-ELISAs using the narrow-specificity MAb M1 proved highly efficient for the detection of TYLCTHV and TbLCYnV, whereas TAS-ELISAs using the broad-specificity MAb D2 were highly efficient for the detection of TYLCTHV, TbLCYnV, ToLCNDV and SLCCNV. Both newly developed assays allow for sensitive, inexpensive, high-throughput detection of begomoviruses in field plant samples, as well as screening for virus-resistant cultivars.

## Background

*Begomovirus* is a genus of plant viruses in the family *Geminiviridae* that have circular single-stranded DNA (ssDNA) genomes encapsidated in a characteristic twinned icosahedral particle [1]. Begomoviruses infect only dicotyledonous plants and are transmitted naturally by whiteflies, *Bemisia tabaci* (Genn.). Typical symptoms of begomovirus infection are leaf curling, leaf yellowing, vein yellowing, mosaic discoloration and stunting of plant growth. Begomoviruses are widespread in tropical and subtropical regions of the world and cause significant yield losses in various economically important crops such as tomatoes, peppers, cucumbers, cotton and cassava [2, 3].

*Tomato yellow leaf curl Thailand virus* (TYLCTHV) is a begomovirus that causes severe losses in tomato production in several countries in East and Southeast Asia [4]. TYLCTHV was first isolated from a tomato production field in Thailand in 1990 [5]. TYLCTHV has a bipartite DNA genome consisting of two circular ssDNA components, referred to as DNA-A and DNA-B, both of which are approximately 2.8 kb in size [6, 7]. Since its complete characterization in 1994, several strains of TYLCTHV have been reported in different regions of Thailand, such as TYLCTHV-[NK] from Nong Khai province, TYLCTHV-[CM] from Chiang Mai and TYLCTHV-[SK] from Sakon Nakhon [4, 8]. TYLCTHV has also been reported to be prevalent in Myanmar, Cambodia, southern China and Taiwan [9–12]. In Taiwan, TYLCTHV has been found to be so virulent that it can overcome the commonly-deployed *Ty-2*-resistant tomato cultivars [10]. Recent studies have shown that TYLCTHV tends to supplant the local *Tomato leaf curl Taiwan virus* (ToLCTV) in many parts of Taiwan [13]. In addition to tomatoes, TYLCTHV has also been identified in alternative host plants, namely hot peppers and sweet peppers, in Thailand and Taiwan [9, 14].

Diagnosis of TYLCTHV and other begomoviruses in their reservoir hosts is necessary for epidemiological study and disease management. Breeding programs for selecting begomovirus-resistant plants also require efficient detection techniques for screening and evaluating resistant cultivars. Several serological and molecular techniques have been developed for the detection and identification of begomoviruses [15–25]. For routine virus detection, enzyme-linked immunosorbent assay (ELISA) has been commonly used, owing to its simplicity, sensitivity, accuracy and affordability. Monoclonal antibodies (MAbs) and polyclonal antibodies (PAbs) against different species of begomoviruses have been developed by using viral antigens from purified virus particles or recombinant virus proteins expressed in the *Escherichia coli* system [26, 27]. These antibodies have been used to develop many ELISA-based methods for the detection of various begomoviruses, such as a double antibody sandwich-ELISA (DAS-ELISA) for the detection of *Potato apical leaf curl virus* (PALCV) [28] and a triple antibody sandwich-ELISA (TAS-ELISA) for the detection of *Tomato yellow leaf curl virus* (TYLCV) [29–32]. Nevertheless, there have been no reports on the development of MAbs and ELISAs for the detection of TYLCTHV to date.

In this study, MAbs to TYLCTHV were generated against recombinant TYLCTHV coat protein expressed in *E. coli* cells. The purified MAbs were characterized and used to develop TAS-ELISAs for begomovirus detection. The efficiency of these newly established TAS-ELISAs in detecting TYLCTHV and other begomoviruses was evaluated with different types of field plant samples collected from different provinces of Thailand. The association between the CP amino acid sequences of begomoviruses identified in this study and their serological reactivity to MAbs was also investigated.

## Methods

### Sources of viruses

TYLCTHV isolate TH:2 (TYLCTHV-[TH:2]) was maintained in tomato plants by graft inoculation and used as a positive control [6]. Other begomoviruses including *Tomato leaf curl New Delhi virus* (ToLCNDV), *Squash leaf curl China virus* (SLCCNV), *Tobacco leaf curl Yunnan virus* (TbLCYnV), *Bhendi yellow vein mosaic virus* (BYVMV), *Pepper yellow leaf curl Indonesia virus* (PepYLCIV) and *Tomato yellow leaf curl Kanchanaburi virus* (TYLCKaV) were previously identified in the authors’ laboratory by PCR amplification of the coat protein (CP) gene using CP5 and CP2 primers (5' ATG TCG AAG CGT CCA GCA GA 3' and 5' TTA ATT CGT CAC TGA GTC AT 3', respectively) and subsequent DNA sequencing and BLAST analysis of the amplified CP gene. Partially purified TYLCTHV, ToLCNDV and SLCCNV were prepared from the corresponding begomovirus-infected plants, according to the method described by Honda et al. [33]. Sap extracts from infected plants as well as partially purified viruses were used for the characterization of MAbs. Sap extracts from plants previously diagnosed for other unrelated plant viruses including *Tobacco mosaic virus* (TMV), *Cucumber mosaic virus* (CMV), *Papaya ringspot virus* (PRSV), *Watermelon mosaic virus-2* (WMV-2), *Watermelon silver mottle virus* (WSMoV), Tomato necrotic ringspot virus (TNRV), Capsicum chlorosis virus (CaCV), Melon yellow spot virus (MYSV), *Cucumber green mottle mosaic virus* (CGMMV) and *Cucurbit aphid-borne yellows virus* (CABYV) were used for specificity analyses of MAbs.

Leaves of plants showing typical symptoms of begomovirus infection (leaf-curling, leaf yellowing, vein yellowing, mosaic discoloration, and stunting) were collected from production fields in several provinces of Thailand: Chiang Mai, Khon Kaen, Nong Khai, Maha Sarakham, Nakhon Pathom, Kanchanaburi, Suphan Buri, Ratchaburi, Petchaburi, and Phang-Nga. Crop plants tested included tomatoes, peppers, wax gourds, cucumbers, melons, loofah, pumpkins, eggplants and okra. Weeds found within or bordering the crop fields, such as goat weed (*Ageratum conyzoides* L.), broom weed (*Sida acuta* Burm.f.), and node weed (*Synedrella nodiflora* L.), were also collected and used for evaluation of TAS-ELISAs.

### Cloning and expression of the TYLCTHV CP gene

The coat protein gene of TYLCTHV-[TH:2] (GenBank accession number: AF141922) was amplified from the full-length cloned DNA of TYLCTHV component A, pTYLCTHVA [6, 24], by PCR using primers CPA1 (5’CGG GAT CCA TGT CGA AGC GTC CAG 3’) and CPA2 (5’ CCC AAG CTT TTA ATT CGT CAC TGA G 3’). The 771-bp PCR product was cloned in-frame into the expression vector pQE30 (QIAGEN, Hilden, Germany) at *Bam*HI and *Hind*III sites, thus yielding plasmid pQE30-TYLCTHV-CP with a 6 × His tag at the N-terminus of the coat protein. Plasmid pQE30-TYLCTHV-CP was transformed into *E. coli* strain M15 containing the repressor (pREP4) plasmid. Transformants carrying recombinant plasmids with the correct insert size were selected by colony PCR. The identities of the transformants were confirmed by plasmid preparation and DNA sequence analysis. Selected clones expressing recombinant coat proteins were grown to an absorbance at 600 nm (OD_600_) of 0.6 at 37 °C in LB medium containing 100 μg/ml ampicillin and 25 μg/ml kanamycin. Protein expression was induced by addition of 1 mM isopropyl-1-thio-β-D galactoside (IPTG) for 5 h at 37 °C. Recombinant TYLCTHV-CP was purified using Ni-NTA agarose resin column (QIAGEN, Hilden, Germany) under denaturing conditions.

Purified proteins were analyzed by western blotting after separation by 12% sodium dodecyl sulfate polyacrylamide gel electrophoresis (SDS-PAGE) and probed with a rabbit polyclonal antibody to begomovirus (kindly provided by Ms. Kruapan Kittipakorn, Department of Agriculture, Thailand). The purified protein was dialyzed against phosphate buffered saline (PBS), pH 7.4, before being used as an immunogen.

### Preparation of hybridomas and MAbs

Six-week-old female BALB/c mice were injected intraperitoneally with the purified recombinant TYLCTHV-CP. Each mouse was subsequently immunized 5 times at 2-week intervals with 100 μg of recombinant TYLCTHV-CP in PBS (pH 7.4) per immunization. Serum from each immunized mouse obtained 7 days after each immunization was tested for antibodies against recombinant TYLCTHV-CP by plate-trapped antigen ELISA (PTA-ELISA). These mouse antisera were also evaluated for the ability to differentiate between TYLCTHV-infected and healthy tomatoes by PTA-ELISA and western blot analysis. The mouse with the highest antibody titer to recombinant TYLCTHV-CP was boosted with 200 μg of the purified recombinant TYLCTHV-CP in PBS (pH 7.4) and sacrificed four days later for hybridoma preparation. Isolated mouse splenic cells were fused with P3x63-Ag8.653 mouse myeloma cells (ratio of 5:1, splenic cells: P3x63-Ag8.653 cells) in the presence of 50% (w/v) polyethylene glycol 4000 (Sigma-Aldrich, St. Louis, MO, USA). Hybridoma cells were selected by culturing in a hypoxanthine-aminopterin-thymidine (HAT) medium. Cultures were propagated and screened for production of antibodies against recombinant TYLCTHV-CP by PTA-ELISA as described below. Hybridoma cells found to produce antibodies against recombinant TYLCTHV-CP were tested against sap extracts from TYLCTHV-infected and healthy tomatoes by PTA-ELISA. Hybridoma cultures that produced antibodies to recombinant TYLCTHV-CP and were able to differentiate between TYLCTHV-infected and healthy tomatoes were subcultured at limiting dilutions to produce monoclonal cultures of antibody-producing hybridoma cells. Large-scale production of MAbs from the selected hybridoma clones was performed in a WHEATON®CELLine^TM^ bioreactor (Wheaton, Millville, NJ, USA).

### PTA-ELISA

PTA-ELISAs for initial screening for antibody activity were carried out as follows. Half of the wells in a 96-well microtiter plate (Costar, Cambridge, MA, USA) were coated for 3 h at 37 °C with 100 μl of 40 μg/ml recombinant TYLCTHV-CP in coating buffer (50 mM carbonate buffer, pH 9.6), and the other half were coated with the coating buffer alone (blank). After four successive washings with 400 μl per well of PBS plus 0.05% Tween 20 (PBST), plates were blocked with 100 μl of 2% (w/v) bovine serum albumin (Sigma-Aldrich, St. Louis, MO, USA) in PBST overnight at 4 °C. After overnight incubation, the plates were washed as described above. Hybridoma culture supernatant (100 μl) was then added to each well and incubated for 2 h at 37 °C. After the plates were washed with PBST, the captured mouse antibodies were detected by addition of 100 μl of alkaline phosphatase-conjugated goat anti-mouse polyvalent immunoglobulin (G, A, M) (Sigma-Aldrich, St. Louis, MO, USA) diluted 1:2000 in 0.5% (w/v) BSA in PBST. The plates were incubated for 1 h at 37 °C and washed with PBST. Substrate for alkaline phosphatase (100 μl of *p*-nitrophenyl phosphate; Life Technologies, NY, USA) was added, and after a 1 h incubation at 37 °C, the reaction was stopped by adding 50 μl of 3 N NaOH. The absorbance at 405 nm (OD_405_) was measured using an automated microplate reader (Multiskan EX, Labsystems, Helsinki, Finland). Prefusion mouse serum was used as a positive control, and HAT medium and medium from the myeloma culture were used as negative controls. A test was considered positive when the absorbance was at least twice that of the negative control.

PTA-ELISA for begomovirus detection in plant sap was performed as follows. Leaves of infected or healthy plants (0.4 g fresh leaf tissue or 0.025 g dried leaf tissue) were homogenized in 1 ml of extraction buffer (0.05 M Tris-HCl; 0.06 M sodium sulfite, pH 8.5). After centrifugation of the samples at 10,000 x g for 5 min, the supernatant was collected. Plates were coated with plant sap extracts diluted two-fold in coating buffer (50 mM carbonate buffer, pH 9.6) and incubated at 4 °C overnight. The assay method was the same as that described above.

### Characterization of MAbs

MAb isotypes were determined using a Mouse Immunoglobulin Isotyping ELISA kit (BD Biosciences, San Diego, CA, USA). The serological reactivity of MAbs was initially characterized by PTA-ELISA and western blot analysis against recombinant TYLCTHV-CP and partially purified begomoviruses, including TYLCTHV, ToLCNDV and SLCCNV. The serological reactivity of MAbs was further determined by PTA-ELISA using sap extracts from plants infected with TYLCTHV, ToLCNDV, SLCCNV, TbLCYnV, BYVMV, PepYLCIV and TYLCKaV. The specificity of MAbs was also determined against the following unrelated plant viruses: TMV, CMV, PRSV, WMV-2, WSMoV, TNRV, CaCV, MYSV, CGMMV and CABYV.

### Western blot analysis

Four micrograms of recombinant TYLCTHV-CP protein, 50 μg of protein extracts from plant sap, or 10 μl of a partially purified virus preparation was subjected to discontinuous SDS-PAGE using 5% stacking and 12% separating gels. Rainbow^TM^ protein molecular weight markers (Amersham, Buckinghamshire, UK) were included in each gel. The separated proteins were transferred to 0.45 μm nitrocellulose membranes (Protran™ GE Healthcare Life Science, Karlsruhe, Germany) by electroelution, and the blotted membranes were air-dried. The following steps were performed on a shaker at room temperature. The membranes were soaked in 5 ml of Tris-buffered saline plus Tween-20 (TBST; 10 mM Tris pH 8.0, 150 mM NaCl, 0.05% Tween 20) for 10 min and subsequently blocked with 5 ml of 4% (w/v) BSA in TBST for 1 h. The membranes were then incubated with 5 ml of MAb M1 (diluted 1/200) or MAb D2 (diluted 1/800) in 1% (w/v) BSA in TBST for 1 h. After three successive washes with TBST (10 min each), the membranes were incubated with alkaline phosphatase-conjugated goat anti-mouse polyvalent immunoglobulin (G, A, M) for 1 h. The membranes were washed as described above and incubated with BCIP/NBT substrate solution (Invitrogen, Waltham, MA, USA) for 5-10 min. Color development was stopped by immersion of the membrane in distilled water.

### TAS-ELISA

Sap extracts were prepared by grinding leaf tissues (1 g) in 5 ml of extraction buffer (0.05 M Tris-HCl; 0.06 M sodium sulfite, pH 8.5). After centrifugation of samples at 10,000 x g for 5 min, the supernatant was collected. Plates were coated with rabbit polyclonal antibody to begomovirus diluted 1:5000 in coating buffer and incubated for overnight at 4 °C. After three successive washes with PBST, plates were blocked with 2% (w/v) BSA in PBST for 1 h at 37 °C. After the plates were washed again, plant sap extracts were then added and incubated for 1 h at 37 °C. The plates were washed, and MAb M1 (diluted 1:200) or MAb D2 (diluted 1:800) in 0.5% (w/v) BSA in PBST was then added, and the plates were incubated for 1 h at 37 °C. The detection steps were the same as those described for PTA-ELISA.

### PCR and DNA analysis of CP genes

Genomic DNA was extracted from leaf samples with a GeneJet Plant Genomic DNA purification kit (Thermo Scientific, Waltham, MA, USA). Universal degenerate primers targeting conserved regions on begomovirus DNA-A (Beg2F: 5’-TAT GBC GAA GCG WBC HRY MGA-3’ and Beg11bR: 5’-TTC AAY YAC AAC CTS MGG ARR G-3’) were designed and used to amplify PCR products of 1022 bp covering the entire CP gene and part of the replication enhancer (REn) gene. PCR reactions were carried out under the following conditions: initial denaturation at 94 °C for 5 min; 35 cycles of PCR at 95 °C for 45 s, 52 °C for 45 s, and 72 °C for 1 min and 15 s; and a final extension at 72 °C for 7 min. PCR products were analyzed by electrophoresis on 1.0% agarose gels in 1× TAE buffer (40 mM Tris, 20 mM acetic acid, and 1 mM EDTA). Amplified fragments from the PCR-positive samples were individually cloned into the pGEM-T Easy Vector (Promega, Madison, WI, USA), according to the manufacturer’s protocol, and transformed into the *E. coli* strain DH5α. For each PCR product, 3 independent clones containing an insert of the expected size were selected for plasmid preparation and were sent for DNA sequencing at 1^st^ BASE Laboratories (Selangor, Malaysia). Nucleotide sequence comparison was then performed with the nucleotide BLAST program (https://blast.ncbi.nlm.nih.gov/Blast.cgi). Multiple sequence alignment of the deduced CP amino acid sequences of begomoviruses recognized and not recognized by MAbs M1 and D2 was performed using Clustal W software from DNASTAR.

## Results

### Production of MAbs against TYLCTHV coat protein

**Fig. 1:**
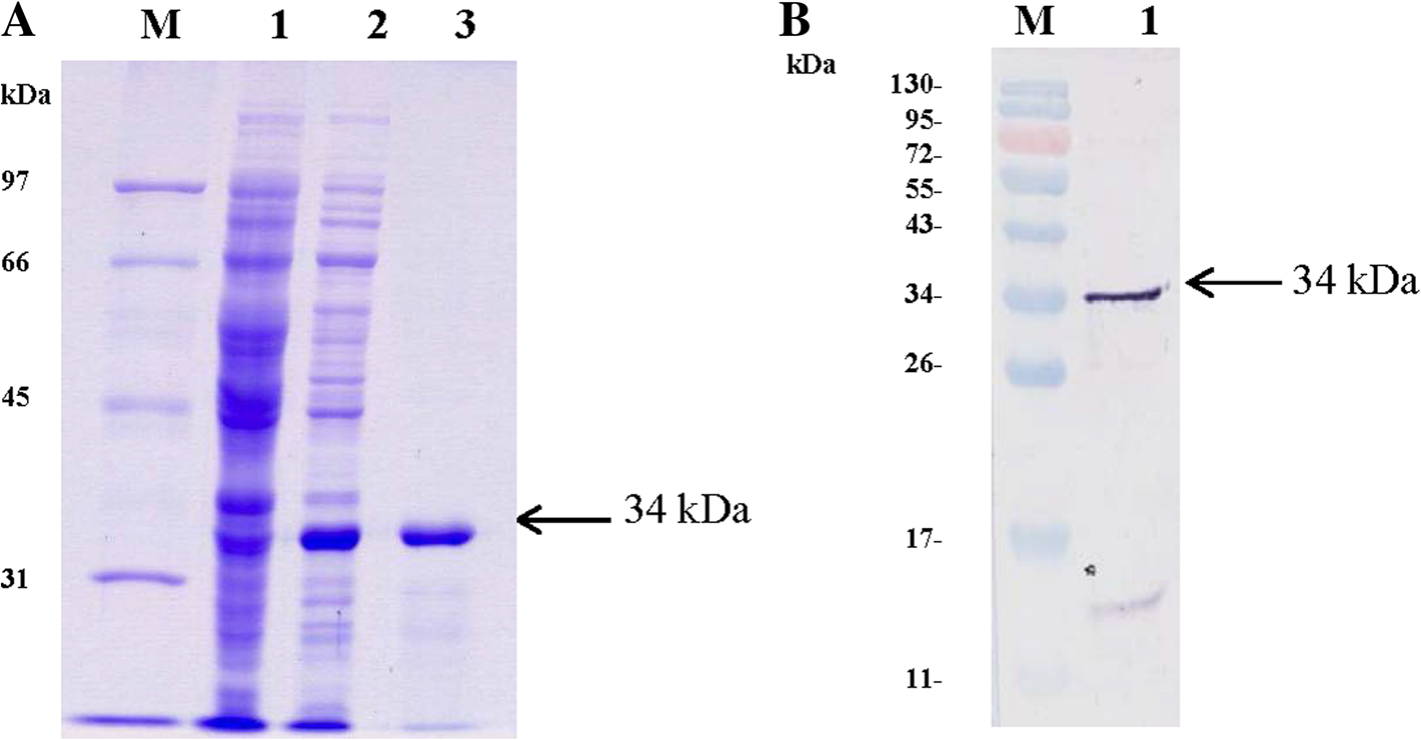
Analyses of the recombinant TYLCTHV-CP protein by SDS-PAGE and western blotting. **a** SDS-PAGE analysis of the recombinant TYLCTHV-CP protein. Lysate of non-induced *E. coli* cells harboring pQE30-TYLCTHV-CP (*Lane 1*); lysate of IPTG-induced *E. coli* cells harboring pQE30-TYLCTHV-CP (*Lane 2*); and the purified recombinant TYLCTHV-CP (*Lane 3*) were analyzed by 12% SDS-PAGE and subsequent Coomassie blue staining. The Low-Range SDS-PAGE Standard (Bio-Rad, Richmond, CA, USA) (*Lane M*) was used as the molecular weight marker. **b** Western blot analysis of the recombinant TYLCTHV-CP. The purified recombinant TYLCTHV-CP protein was analyzed by western blotting using a rabbit polyclonal antibody to begomovirus (*Lane 1*). The Rainbow^TM^ protein molecular weight marker was included (*Lane M*)

To produce MAbs against TYLCTHV, the full-length CP ORF (771 bp) of TYLCTHV-[TH:2] was expressed in *E. coli* as a histidine-tagged fusion protein (TYLCTHV-CP) in response to IPTG induction, thus yielding a 34-kDa protein band detected through 12% SDS-PAGE and Coomassie blue staining (Fig. 1a). Recombinant TYLCTHV-CP in the insoluble fraction of the cell homogenate was purified using an Ni-NTA agarose resin column under denaturing conditions, yielding approximately 20 mg of purified TYLCTHV-CP per liter of bacterial culture. The purified recombinant TYLCTHV-CP was confirmed by western blot analysis using a PAb to begomovirus. As shown in Fig. 1b, a 34-kDa band was visualized in Lane 1, thus indicating that recombinant TYLCTHV-CP was successfully expressed and purified. The faint band (14 kDa) observed in Lane 1 appeared to be a degradation fragment of the purified TYLCTHV CP protein. The purified TYLCTHV-CP was then used to immunize BALB/c mice for antibody production.

The mouse showing the highest antibody titer against the TYLCTHV-CP protein (1:64,000 by PTA-ELISA) was selected for hybridoma preparation. Among 480 fusion wells, 267 contained growing hybridoma cells (55.6%). Among those, 11 showed positive reactivity to the purified TYLCTHV-CP protein by PTA-ELISA (OD_405_ = 0.391–1.373) (data not shown). After three successive rounds of subcloning, two stable hybridoma cell lines secreting MAbs M1 and D2 were identified and selected for further characterization.

### Characterization of MAbs by western blot and PTA-ELISA

**Fig. 2:**
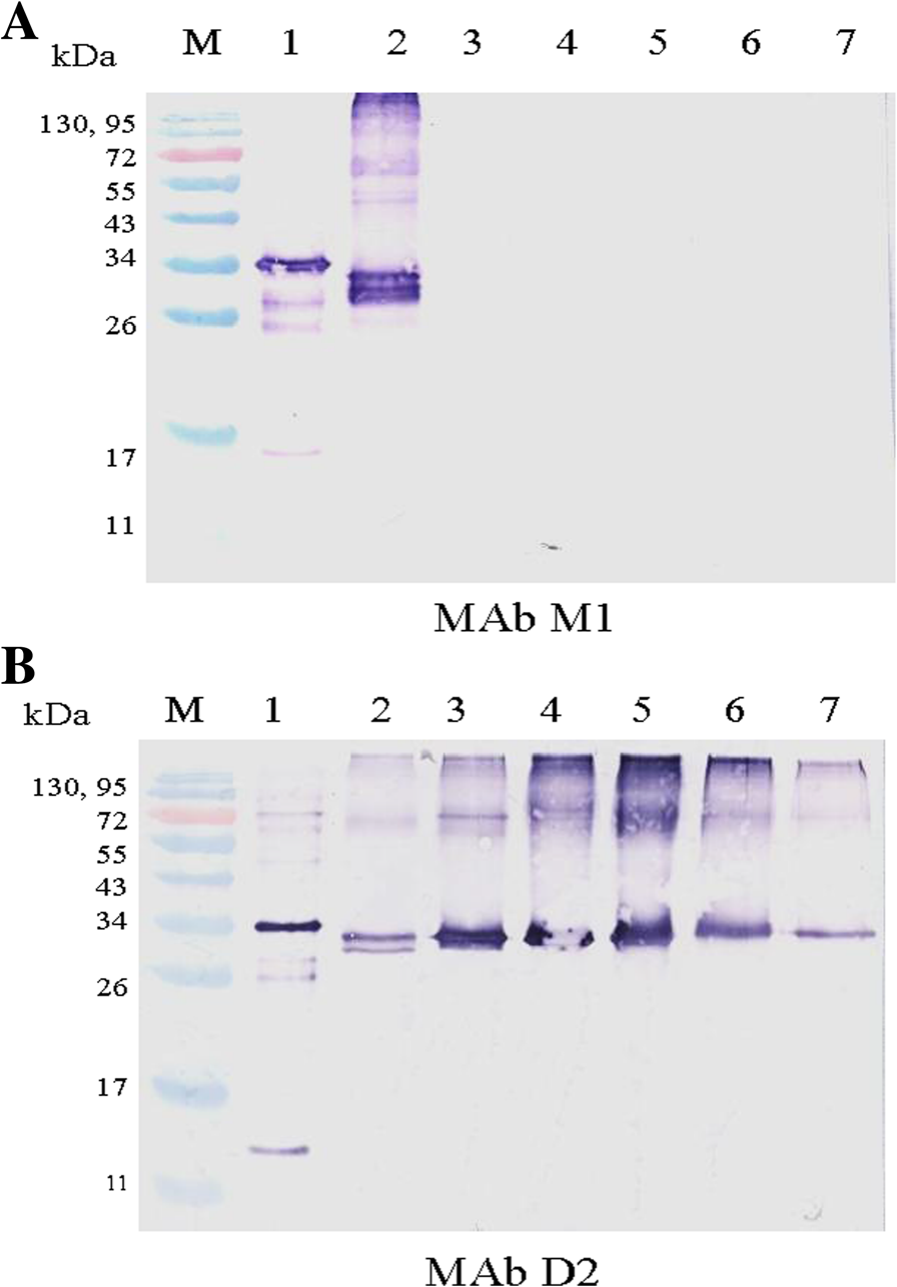
Analysis of serological reactivity of MAbs M1 and D2 by western blotting. The recombinant TYLCTHV-CP protein, partially purified TYLCTHV, partially purified ToLCNDV and partially purified SLCCNV from various plants were subjected to western blotting by probing with MAbs M1 (**a**) and D2 (**b**). *Lane 1*: recombinant TYLCTHV-CP protein; *Lane 2*: partially purified TYLTHCV from tomatoes; *Lane 3*: partially purified ToLCNDV from cucumbers; *Lane 4*: partially purified SLCCNV from ivy gourds; *Lane 5*: partially purified SLCCNV from pumpkins; *Lane 6*: partially purified SLCCNV from wax gourds; *Lane 7*: partially purified ToLCNDV from loofahs; *Lane M*: Rainbow^TM^ protein molecular weight marker

The immunoglobulin classes and subclasses of MAbs M1 and D2 were isotyped as IgG1 and IgG2b, respectively. The light chains of both MAbs were of the kappa light chain type. Serological reactivity of MAbs M1 and D2, as determined by western blot analysis, showed that MAb M1 reacted strongly with recombinant TYLCTHV-CP protein and partially purified TYLCTHV from infected tomatoes (molecular weights of approximately 34 and 33 kDa, respectively; Fig. 2a). No serological reactivity was observed between MAb M1 and partially purified ToLCNDV or SLCCNV. MAb D2, however, reacted not only with recombinant TYLCTHV-CP protein and partially purified TYLCTHV but also with partially purified ToLCNDV and SLCCNV (Fig. 2b).

The serological reactivity of MAbs M1 and D2 was further investigated by PTA-ELISA against recombinant TYLCTHV-CP protein, partially purified TYLCTHV, ToLCNDV and SLCCNV, and sap extracts from TYLCTHV-, ToLCNDV-, SLCCNV-, TbLCYnV-, BYVMV-, PepYLCIV and TYLCKaV-infected leaves. As summarized in Table 1, MAb M1 reacted strongly with recombinant TYLCTHV-CP protein, partially purified TYLCTHV, and sap extracts from TYLCTHV- and TbLCYnV-infected plants, but not with sap extracts from ToLCNDV-, SLCCNV-, BYVMV-, PepYLCIV and TYLCKaV-infected tissues. MAb D2 reacted with a broader range of begomovirus species, including TYLCTHV, TbLCYnV, ToLCNDV, and SLCCNV, but not with BYVMV, PepYLCIV, or TYLCKaV. No serological reactivity was observed with healthy plant controls or the unrelated plant viruses tested, i.e., TMV, CMV, PRSV, WMV-2, WSMoV, TNRV, CaCV, MYSV, CGMMV or CABYV.

**Table 1:** Specificity analysis of MAbs M1 and D2 against begomoviruses and other plant viruses by PTA-ELISA

Antigens	PTA-ELISA (Absorbance 405 nm)	
	MAb M1	MAb D2
Recombinant TYLCTHV-CP protein	**1.495**	**3.274**
Partially purified viruses:		
TYLCTHV from tomato	**1.800**	**2.399**
ToLCNDV from cucumber	0.110	**2.648**
SLCCNV from ivy gourd	0.107	**0.711**
SLCCNV from pumpkin	0.081	**2.049**
SLCCNV from wax gourd	0.086	**1.788**
ToLCNDV from loofah	0.101	**2.944**
Coating buffer	0.106	0.096
Sap extracts:		
TYLCTHV-infected tomato	**1.262**	**0.928**
ToLCNDV-infected cucumber	0.091	**2.442**
SLCCNV-infected pumpkin	0.126	**1.783**
SLCCNV-infected wax gourd	0.121	**1.424**
ToLCNDV-infected loofah	0.111	**0.638**
TbLCYnV-infected tomato	**0.375**	**0.534**
BYVMV-infected okra	0.110	0.104
PepYLCIV-infected pepper	0.120	0.105
TYLCKaV-infected eggplant	0.130	0.100
TMV-infected leaves	0.118	0.173
CMV-infected leaves	0.124	0.189
PRSV-infected pumpkin	0.114	0.184
WMV-2-infected leaves	0.115	0.171
WSMoV-infected *Physalis minima*	0.124	0.184
CaCV-infected *Physalis minima*	0.106	0.153
MYSV-infected *Physalis minima*	0.111	0.167
TNRV-infected *Physalis minima*	0.120	0.183
CGMMV-infected cucumber	0.122	0.191
CABYV-infected bitter gourd	0.113	0.157
Healthy tomato	0.093	0.111
Healthy cucumber	0.116	0.171
Healthy pumpkin	0.108	0.160
Healthy wax gourd	0.083	0.103
Healthy loofah	0.121	0.161
Healthy bitter gourd	0.112	0.175

Cut-off value = two times the absorbance (OD_405_) of the healthy control

Bold letter indicates ODs higher than the associated cut-off value

Both the western blot analysis and PTA-ELISA results suggested that MAbs M1 and D2 are specific for begomoviruses. MAb M1 was thus suitable to detect 2 begomoviruses (TYLCTHV and TbLCYnV). MAb D2 demonstrated broader serological reactivity and was thus suitable to detect 4 begomoviruses, including TYLCTHV, TbLCYnV, ToLCNDV and SLCCNV.

### TAS-ELISA for begomovirus detection

Two TAS-ELISAs were developed using a PAb against begomovirus (diluted 1:5000 in coating buffer) as the capture Ab and the newly established MAb M1 (diluted 1:200 in 0.5% (w/v) BSA in PBST) or D2 (diluted 1:800 in 0.5% (w/v) BSA in PBST) as detection Abs.

**Fig. 3:**
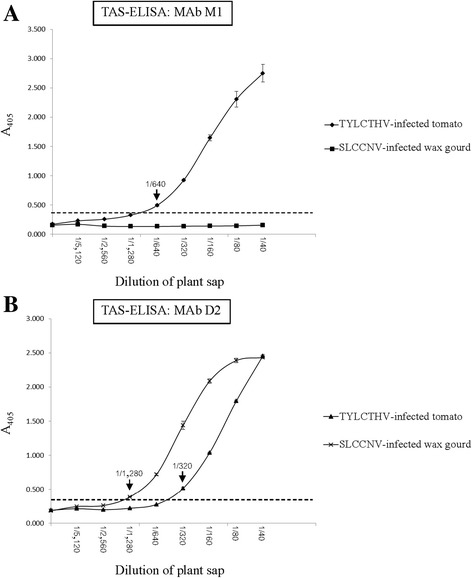
Sensitivity analyses of TAS-ELISA using MAbs M1 and D2 for begomovirus detection. Sap extracts from TYLCTHV-infected tomatoes and SLCCNV-infected wax gourds were two-fold serially diluted in sap extracts from healthy tomatoes or healthy wax gourds from 1:40 to 1:5120 (w/v, g/ml) and used to determine the sensitivity of MAb M1- (**a**) or D2- (**b**) based TAS-ELISAs. *Dashed lines* indicate the cut-off values for the TAS-ELISAs (twice the OD_405_ of healthy controls)

To investigate the sensitivity of both TAS-ELISA systems, sap extracts prepared from TYLCTHV-infected tomato leaf tissues and SLCCNV-infected wax gourd leaf tissues were two-fold serially diluted and subjected to begomovirus detection by TAS-ELISA using MAbs M1 and D2 (Fig. 3). The results showed that TAS-ELISA using MAb M1 was able to detect TYLCTHV in infected tomato leaf extracts diluted up to 1:640 (Fig. 3a). TAS-ELISA using MAb D2 was able to detect TYLCTHV in infected tomato leaf extracts diluted up to 1:320 and SLCCNV in infected gourd leaf extracts diluted up to 1:1280 (Fig. 3b). These results suggested that the newly developed TAS-ELISAs using narrow-specificity MAb M1 or broad-specificity MAb D2 were highly sensitive for the detection of TYLCTHV or TYLCTHV and SLCCNV in infected leaf samples, respectively.

### Applying TAS-ELISAs to TYLCTHV and begomovirus detection in field samples

To determine the usefulness of the newly developed TAS-ELISAs for field samples, a total of 147 leaf samples with begomovirus-like symptoms were collected from tomato, pepper, cucumber, eggplant, loofah, melon, pumpkin, wax gourd, okra, and ivy gourd crops in different provinces of Thailand. These samples were tested for begomovirus infection by TAS-ELISA using MAbs M1 and D2, as well as by PCR using universal degenerate primers for the begomovirus CP gene. Among the 147 samples, 30 tested negative by both TAS-ELISAs and PCR and were thus considered to be negative for begomovirus infection (Table 2). The remaining 117 samples tested positive by PCR; among these, 73 tested positive by TAS-ELISA using either MAbs M1 and/or D2. The PCR products of all 117 PCR-positive samples were cloned and sequenced to determine the species of begomoviruses from both ELISA-positive and ELISA-negative samples. DNA sequencing and BLAST analysis of the CP genes identified 9 distinct begomovirus species from the 117 PCR-positive samples: TYLCTHV, ToLCNDV, SLCCNV, TbLCYnV, BYVMV, TYLCKaV, PepYLCIV, *Ageratum yellow vein virus* (AYVV), and *Pepper leaf curl virus* (PepLCV).

**Table 2:** Detection and identification of begomoviruses in field samples by TAS-ELISA, PCR and DNA sequence analysis

Host	No. of samples tested	No. of samples positive by PCR	TAS-ELISA^e^		Species based on sequence analysis of cp gene								
			No. of samples positive by M1	No. of samples positive by D2	TYLCTHV	TbLCYnV	ToLCNDV	SLCCNV	PepLCV	AYVV	BYVMV	PepYLCIV	TYLCKaV
Tomato	37	27	15	21	11^a^	4^a^	1^b^			9^(5c,4d)^			2^d^
Cucumber	39	29	0	29			29^b^						
Loofah	6	1	0	1			1^b^						
Ivy gourd	1	1	0	1			1^b^						
Melon	9	4	0	4			2^b^	2^b^					
Pumpkin	5	5	0	5				5^b^					
Wax gourd	2	2	0	2				2^b^					
Eggplant	5	5	0	0									5^d^
Okra	5	5	0	0							5^d^		
Pepper	34	34	0	9					24^(9c,15d)^			8^d^	2^d^
Weed	4	4	1	1		1^a^			1^d^	2^d^			
Total	147	117	15	73	11	5	34	9	25	11	5	8	9

^a^Positive with M1 and D2

^b^Positive with D2

^c^Weakly positive with M1 and/or D2

^d^Negative with M1 and D2

^e^Sample positive by MAb M1 was also positive by MAb D2

As summarized in Table 2, our results demonstrated that all of the TYLCTHV-infected samples (11 tomato samples) and TbLCYnV-infected samples (4 tomato samples, 1 goat weed sample) tested positive by both MAbs M1 and D2. All of the ToLCNDV-infected samples (29 cucumber, 2 melon, 1 loofah, 1 ivy gourd, 1 tomato) and SLCCNV-infected samples (5 pumpkin, 2 melon, 2 wax gourd samples) tested positive by MAb D2 but not M1. None of the BYVMV-infected okra samples (5), PepYLCIV-infected pepper samples (8) or TYLCKaV-infected samples (5 eggplant, 2 tomato, 2 pepper samples) showed positive results with either MAb M1 or D2. Of 25 PepLCV-infected samples (24 pepper, 1 node weed samples), only 9 (peppers) showed weakly positive results with MAb M1 and/or D2. For AYVV-infected samples (9 tomato, 2 broom weed samples), only 5 (tomatoes) showed weakly positive results with MAb M1 and/or D2. Our studies on various types of field-collected plant samples indicated that TAS-ELISA using MAb M1 was able to efficiently detect TYLCTHV and TbLCYnV, whereas TAS-ELISA using MAb D2 was able to efficiently detect TYLCTHV, TbLCYnV, ToLCNDV and SLCCNV. Neither MAb M1 nor D2 showed positive results with BYVMV, TYLCKaV or PepYLCIV. Several of the PepLCV- and AYVV-infected samples showed mildly positive results with MAbs M1 and/or D2.

### Association between the deduced CP amino acid sequences of begomoviruses and their serological reactivity to MAbs M1 and D2

**Fig. 4:**
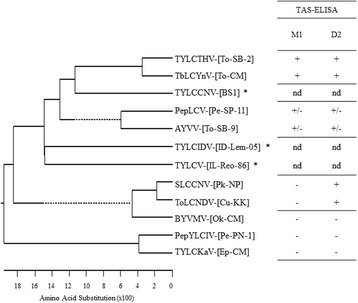
Phylogenetic tree showing the relationships between the deduced CP amino acid sequences of begomovirus species identified in this study. The deduced CP amino acid sequences of 9 begomovirus isolates representing 9 begomovirus species, namely, TYLCTHV-[To-SB-2], TbLCYnV-[To-CM], PepLCV-[Pe-SP-11], AYVV-[To-SB-9], ToLCNDV-[Cu-KK], SLCCNV-[Pk-NP], BYVMV-[Ok-CM], PepYLCIV-[Pe-PN-1], and TYLCKaV-[Ep-CM], were aligned with CP amino acid sequences of tomato yellow leaf curl begomoviruses that have been reported in East and Southeast Asia, but not in Thailand, including *Tomato yellow leaf curl virus* (TYLCV) X15656, *Tomato yellow leaf curl China virus* (TYLCCNV) AJ319675, and *Tomato yellow leaf curl Indonesia virus* (TYLCIDV) AF189018. *Asterisks* indicate the related tomato yellow leaf curl begomoviruses for phylogenetic comparison

The results of begomovirus detection in field samples demonstrated that MAbs M1 and D2 differed in their serological reactivity. MAb M1 demonstrated a narrow spectrum of serological reactivity, detecting only TYLCTHV and TbLCYnV, whereas MAb D2 demonstrated broader serological reactivity, binding not only TYLCTHV and TbLCYnV but also ToLCNDV and SLCCNV. The deduced CP amino acid sequences of the 9 begomovirus species identified in this study were aligned with CP amino acid sequences of the related tomato yellow leaf curl begomoviruses that have been reported in East and Southeast Asia, but not in Thailand, i.e. *Tomato yellow leaf curl virus* (TYLCV) X15656, *Tomato yellow leaf curl China virus* (TYLCCNV) AJ319675, and *Tomato yellow leaf curl Indonesia virus* (TYLCIDV) AF189018 (Table 3). This multiple alignment revealed that CP amino acid sequences of begomoviruses recognized by the narrow-spectrum MAb M1 (TYLCTHV and TbLCYnV) were highly conserved, sharing 93% identity with each other and only 72–81% identity with those that could not be recognized by MAb M1 (ToLCNDV, SLCCNV, BYVMV, PepYLCIV and TYLCKaV). CP amino acid sequences of begomoviruses recognized by the broad-spectrum MAb D2 (TYLCTHV, TbLCYnV, SLCCNV and ToLCNDV) demonstrated a wider range of amino acid sequence identity, sharing 78–96% identity with each other and 72–91% identity with CP of those that could not be detected by MAb D2 (BYVMV, PepLCIV and TYLCKaV). CP amino acid sequences of PepLCV and AYVV, both of which showed weakly positive ELISA results in less than 50% of the virus-infected samples, shared 81–85% identity with the CP amino acid sequences of M1- and/or D2-positive begomoviruses (TYLCTHV, TbLCYnV, SLCCNV, and ToLCNDV) (Table 4). CP amino acid sequences of TYLCCNV, TYLCIDV, and TYLCV (none of which have been reported in Thailand) shared 80-81, 74-75 and 73-74% identity, respectively, with those of M1-positive begomoviruses (TYLCTHV and TbLCYnV) and shared 77-81, 74-78 and 73-76% identity, respectively, with those of D2-positive begomoviruses (TYLCTHV, TbLCYnV, ToLCNDV and SLCCNV). Figure 4 depicts the phylogenetic relationships between the CP amino acid sequences of begomoviruses identified in this study and those previously reported in the database.

**Table 3:** CP amino acid sequences of begomoviruses used for phylogenetic analysis

Begomovirus species-[isolate]	Serological reactivity		Host	Location	GenBank Accession no.
	M1	D2			
Begomoviruses identified in this study					
TYLCTHV-[To-SB-2]	+	+	Tomato	Saraburi	KX900496
TbLCYnV-[To-CM]	+	+	Tomato	Chiang Mai	KX900493
PepLCV-[Pe-SP-11]	±	±	Pepper	Suphan Buri	KX900490
AYVV-[To-SB-9]	±	±	Tomato	Saraburi	KX900488
ToLCNDV-[Cu-KK]	-	+	Cucumber	Khon Kaen	KX900494
SLCCNV-[Pk-NP]	-	+	Pumpkin	Nakhon Pathom	KX900492
BYVMV-[Ok-CM]	-	-	Okra	Chiang Mai	KX900489
PepYLCIV-[Pe-PN-1]	-	-	Pepper	Phang Nga	KX900491
TYLCKaV-[Ep-CM]	-	-	Eggplant	Chiang Mai	KX900495
Previously reported tomato yellow leaf curl begomoviruses					
TYLCCNV-[BS1]*	nd	nd	Tomato	China:Yunnan	AJ319675
TYLCIDV-[ID-Lem-05]*	nd	nd	Tomato	Indonesia:Lembang	AF189018
TYLCV-[IL-Reo-86]*	nd	nd	Tomato	Israel	X15656

*Abbreviations*: *TYLCTHV* Tomato yellow leaf curl Thailand virus, *TbLCYnV* Tobacco leaf curl Yunnan virus, *PepLCV* Pepper leaf curl virus, *AYVV* Agerratum yellow vein virus, *SLCCNV* Squash leaf curl China virus, *ToLCNDV* Tomato leaf curl New Delhi virus, *BYVMV* Bhendi yellow vein mosaic virus, *PepYLCIV* Pepper yellow leaf curl Indonesia virus, *TYLCKaV* Tomato yellow leaf curl Kanchanaburi virus, *TYLCCNV* Tomato yellow leaf curl China virus, *TYLCIDV* Tomato yellow leaf curl Indonesia virus, *TYLCV* Tomato yellow leaf curl virus, *nd* not determined. Asterisks indicate the related tomato yellow leaf curl begomovirus species for phylogenetic comparison

**Table 4:** Percentage identity between CP amino acid sequences of begomoviruses with different immunoreactivity to MAbs M1 and D2

Begomovirus	Immuno-reactivity TAS-ELISA		Sequence identity between CP amino acid (%)											
			TYLCTHV-[To-SB-2]	TbLCYnV-[To-CM]	PepLCV-[Pe-SP-11]	AYVV-[To-SB-9]	SLCCNV-[Pk-NP]	ToLCNDV-[Cu-KK]	BYVMV-[Ok-CM]	PepYLCIV-[Pe-PN-1]	TYLCKaV-[Ep-CM]	TYLCCNV-[BS-1]*	TYLCIDV-[Lem-05]*	TYLCV-[IL-Rep-86]*
	M1	D2												
TYLCTHV-[To-SB-2]	+	+		93	81	81	79	78	78	72	72	81	75	73
TbLCYnV-[To-CM]	+	+			81	83	81	81	80	73	73	80	74	74
PepLCV-[Pe-SP-11]	±	±				89	82	81	83	75	75	82	78	78
AYVV-[To-SB-9]	±	±					84	85	84	76	76	83	79	79
SLCCNV-[Pk-NP]	-	+						96	91	73	72	77	77	76
ToLCNDV-[Cu-KK]	-	+							91	74	73	77	78	76
BYVMV-[Ok-CM]	-	-								74	75	78	77	75
PepYLCIV-[Pe-PN-1]	-	-									92	73	72	71
TYLCKaV-[Ep-CM]	-	-										73	71	69
TYLCCNV-[BS-1]*	nd	nd											76	77
TYLCIDV-[Lem-05]*	nd	nd												76
TYLCV-[IL-Rep-86]*	nd	nd												

Asterisks indicate the related tomato yellow leaf curl begomovirus species for phylogenetic comparison. *nd* not determined

## Discussion

The ability to detect begomoviruses in economically important crops and in their whitefly vectors is an essential prerequisite for epidemiological study and disease management. Currently, ELISA is one of the most widely used tools for reliable high-throughput detection of begomoviruses. This report describes the production of MAbs against TYLCTHV—a bipartite tomato-infecting begomovirus that causes severe losses of tomato crops in Thailand and several countries in Southeast and East Asia—as well as the establishment and evaluation of TAS-ELISAs for begomovirus detection using these newly established MAbs.

For the production of MAbs, recombinant TYLCTHV-CP expressed in *E. coli* cells was chosen as the immunogen, because it was relatively easier to produce and purify in large quantities, as compared with the preparation of purified virions. The use of recombinant protein instead of purified virus also eliminated the generation of non-specific MAbs raised against plant protein contaminants in virus preparations [26]. Initial MAb characterization demonstrated that both M1 and D2 MAbs were able to clearly differentiate begomovirus-infected plant samples from healthy or non-begomovirus-infected plant samples, and MAb D2 demonstrated a broader spectrum of recognition than MAb M1.

In this study, TAS-ELISA, one of the most widely used antibody-based methods, was selected for the development of begomovirus detection assays. With this system, MAb M1 or D2 bound to virus particles specifically captured by the PAb to begomovirus pre-coated on the ELISA plate, thus eliminating the challenges associated with PTA-ELISA platforms, such as the virus binding interference caused by components in the plant sap directly coated on the ELISA well [34]. TAS-ELISA using MAbs M1 and D2 proved to be very efficient for begomovirus detection in field samples. TAS-ELISA with MAb D2 detected all TYLCTHV, TbLCYnV, ToLCNDV and SLCCNV samples identified by PCR, demonstrating 100% sensitivity for these four species in samples from all studied plant sources. TAS-ELISA with MAb M1 exhibited narrower specificity, with 100% sensitivity for TYLCTHV and the closely related TbLCYnV. No false positives were observed in begomovirus detection by these two TAS-ELISAs.

MAbs with narrow and broad spectrum specification for begomoviruses, such as MAbs M1 and D2, have previously been reported [35–39]. On the basis of the high conservation of begomovirus CP amino acid sequences [40], it was not surprising that MAbs M1 and D2 cross-reacted with other begomoviruses in addition to TYLCTHV. To understand this cross-reactivity, we analyzed the percentage sequence-identity between CP amino acid sequences of begomoviruses that were positive or negative for MAbs M1 and D2. Phylogenetic analysis of the deduced CP amino acid sequences of begomoviruses revealed several interesting relationships with possible relevance to their reactivity to MAb M1. CP amino acid sequences of M1-positive begomoviruses (TYLCTHV and TbLCYnV) were highly conserved, sharing 93% identity with each other but only 72–81% identity with that of M1-negative begomoviruses (ToLCNDV, SLCCNV, BYVMV, PepYLCIV and TYLCKaV), thus suggesting that the epitopes recognized by MAb M1 may be relevant to the conserved CP amino acid sequences between TYLCTHV and TbLCYnV. CP amino acid sequences of D2-positive begomoviruses (TYLCTHV, TbLCYnV, ToLCNDV and SLCCNV) shared 78–96% identity with each other; however, they also shared 72–91% identity with the CP sequences of D2-negative begomoviruses (BYVMV, PepLCIV and TYLCKaV). Thus, the CP amino acid sequences of D2-positive begomoviruses might not be sufficient to explain the reactivity profile of MAb D2. Other characteristics that should be taken into account include secondary structure, antigenicity, and hydrophilicity profile, as well as three-dimensional structures of conformation epitopes. CP amino acid sequences of PepLCV and AYVV shared 81–85% identity with those of the M1- or D2-positive begomoviruses; nevertheless, positive results with weak serological reactivity to MAbs M1 and D2 were observed in only 45% of PepLCV and AYVV-infected samples. The weak and inconsistent reactions observed with several infected samples may have been due to relatively low affinity/efficiency MAb binding for the epitopes present on the CP of PepLCV and AYVV, which may be slightly different from the epitopes presented on begomoviruses that strongly react with MAbs M1 and D2. CP amino acid sequences of TYLCCNV, TYLCIDV, and TYLCV (none of which have been reported in Thailand) shared 73-81% identity with those of M1- and D2-positive begomoviruses. Whether these related tomato yellow leaf curl begomoviruses can be recognized by MAb M1 and D2 need to be further investigated.

The data presented herein describe the first report of the development of MAbs and TAS-ELISAs for the detection of TYLCTHV. Although the newly developed MAbs M1 and D2 are not exclusively specific to TYLCTHV, owing to the high conservation of CP among begomoviruses, they were found to be very efficient for high-throughput begomovirus detection in field samples. Both MAbs could also be used in combination to help narrow down the identity of begomovirus infections in certain cases. For example, in our study, all TYLCTHV- and TbLCYnV-infected tomatoes yielded positive results with both MAbs M1 and D2 (M1+/D2+ serological pattern), whereas ToLCNDV- and SLCCNV-infected cucurbits showed an M1-/D2+ serological pattern in all samples tested. Currently, our newly developed TAS-ELISAs using MAbs M1 and D2 have already been used by both governmental and private sectors for field inspections, as well as for the selection of virus-resistant plants in breeding programs in Thailand. In addition to virus detection in plant samples, our MAbs can also detect TYLCTHV in viruliferous whiteflies in both TAS-ELISA and western blot detection platforms (data not shown).

Finally, we expect that information regarding the serological reactivity of MAbs M1 and D2, as well as the amino acid sequence analyses of begomovirus CP proteins obtained from this study, should be useful in selecting strategies for the production of novel MAbs that can specifically or broadly detect all begomoviruses prevalent in Thailand and other endemic areas.

## Conclusions

MAb M1 and MAb D2, with narrow-spectrum and broad-spectrum serological reactivity, respectively, to begomoviruses were produced and successfully used to develop TAS-ELISAs for begomovirus detection. The information reported herein demonstrated the high efficiency of the detection of TYLCTHV and TbLCYnV by MAb M1 and TYLCTHV, TbLCYnV, ToLCNDV and SLCCNV by MAb D2. Both TAS-ELISAs are suitable for the rapid, sensitive, accurate, inexpensive and high-throughput detection of begomoviruses in various types of field plant samples. The availability of MAbs targeting begomoviruses, as well as information on their serological reactivity, should prove highly useful in selecting strategies for the production of MAbs that can cover all begomoviruses prevalent in particular endemic areas.
